# Cancer-Induced Resting Sinus Tachycardia: An Overlooked Clinical Diagnosis

**DOI:** 10.3389/or.2024.1439415

**Published:** 2024-08-02

**Authors:** Minas Sakellakis, Jashan Reet, Michail Kladas, Gregory Hoge, Athanasios Chalkias, Miroslav Radulovic

**Affiliations:** ^1^ Department of Medicine, Jacobi Medical Center/North Central Bronx Hospital, Albert Einstein College of Medicine, Bronx, NY, United States; ^2^ Institute for Translational Medicine and Therapeutics, University of Pennsylvania Perelman School of Medicine, Philadelphia, PA, United States; ^3^ Outcomes Research Consortium, Cleveland, OH, United States

**Keywords:** sinus, tachycardia, cancer, diagnosis, prognosis

## Abstract

Elevated resting heart rate is frequently observed in cancer patients, and is associated with increased mortality. Although specific chemotherapeutic agents can induce cardiotoxicity, the presence of sinus tachycardia in chemotherapy-naive patients suggests other factors likely contribute to this clinical presentation. Despite its prevalence, cancer-associated resting sinus tachycardia has not been fully recognized and comprehensively described as a separate clinical entity. Secondary effects of cancer, especially structural cardiac changes, secretory factors (inflammatory cytokines), and thromboembolic disease can cause resting tachycardia. Alternatively, rapid heart rate may reflect compensatory mechanisms responding to increased metabolic demands, raised cardiac output states, and even pain. Hence, cancer-associated tachycardia presents a clinical dilemma; acute life-threatening conditions (such as sepsis, pulmonary embolism, etc.) must be ruled out, but cancer itself can explain resting sinus tachycardia and more conservative management can avoid unnecessary testing, cost and patient stress. Furthermore, identification and management of cardiac conditions associated with cancer may improve survival and the quality of life of cancer patients.

## Introduction

Although cancer initially starts as a localized disease, as it progresses towards the development of metastases, it becomes a systemic disease affecting several organ systems. Elevated resting heart rate is a frequent finding in patients with advanced cancer and is associated with increased mortality (Table 1) [1–3]. Although it is well known that specific chemotherapeutic agents can induce cardiotoxicity, the presence of sinus tachycardia in patients who did not receive chemotherapy suggests other factors also contribute to this clinical presentation [1]. Sinus tachycardia can initially be mild, but as disease progresses the resting heart rate can increase significantly (e.g., 110–130 per minute) [3, 4]. On the other hand, *Anker et al.* found that resting heart rate was not related to cancer stage, hence terminal disease is not a prerequisite for resting sinus tachycardia [4].

**TABLE 1:** Clinical studies on resting sinus tachycardia and clinical outcomes in cancer patients.

Study	Hemu et al. [1]	Park et al. [2]	Anker et al. [3]	Anker et al. [4]
Cancer type	All cancers	Colorectal cancer	Colorectal, pancreatic, non small cell lung cancer	All cancers
Study design	Case control	Case control	Prospective	Prospective
Follow up	10 years	8 years	27 months	25 months
Number of patients RST/no RST	51/571	75/225	87/58	163/385
Cut-off b.p.m. for RST	>100/<100	>81/<81	>75/<75	>90/<90
Major study findings	HR 2.9 for overall mortality	HR 6.183 for advanced adenoma recurrence	HR 1.84 for overall survival	HR 2 for overall mortality

Abbreviations: RST, resting sinus tachycardia; HR, hazard ratio.

Despite its prevalence, cancer-induced sinus tachycardia has not been comprehensively described as a separate clinical diagnosis. As a result, considerable confusion still exists among clinicians regarding its existence and pathogenesis. Also, it is frequently uncertain in clinical practice whether there is a need to investigate other potential culprits of sinus tachycardia in these patients, such as an underlying sepsis, hemorrhage, pulmonary embolism, etc. Although other causes of sinus tachycardia that need immediate attention must be ruled out, cancer by itself can explain the condition and frequently further testing may not be indicated [1, 4].

In the present article, we review potential factors that contribute to cancer-associated sinus tachycardia in order to provide the foundation for optimal diagnosis, workup and management in this vulnerable group of patients.

## Changes in Cardiac Output States

**FIGURE 1:**
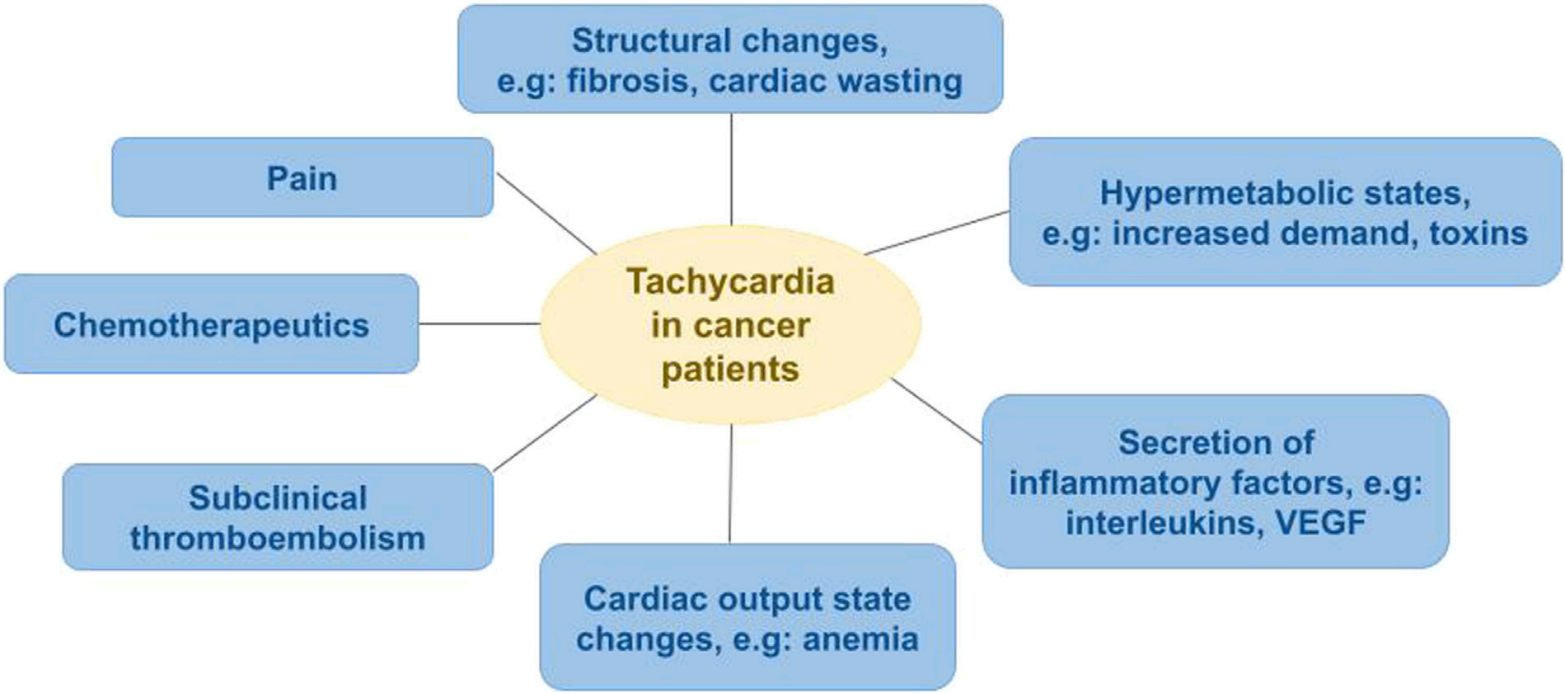
Pathophysiological mechanisms of resting sinus tachycardia in cancer patients.

Many patients with underlying cancer are frequently in high cardiac output states [2]. The potential pathophysiology may be related to cancer-associated anemia or antidiuretic hormone secretion [2, 5]. Studies have shown that patients with cancer frequently have elevated blood pressure, cardiac output, and maximal pressure rise rate during isovolumic contraction, compared to healthy controls and patients with heart failure [2] (Table 2) (Figure 1). In a recent prospective cohort study by Labib *et al.*, 381 patients with chemotherapy-naive lymphoma or active breast cancer, as well as 102 healthy controls underwent standardized cardiovascular magnetic resonance imaging with chamber-volume quantification, native myocardial T1 mapping, and ejection fraction estimation. Although the left ventricular (LV) ejection fraction was similar, cancer patients had significantly increased strain amplitude, systolic strain rate, smaller chambers and native T1, compared to healthy sex-matched controls. These findings persisted after adjusting for age, sex, diabetes mellitus, and hypertension [6]. Cancer patients also had significantly higher right ventricular ejection fraction compared to controls, with mean absolute differences of 3.2% and 1.9% in men and women, respectively. Although cancer patients had smaller left ventricular (LV), right ventricular (RV) and left atrial (LA) volume, as well as, smaller stroke volume, this was successfully compensated for by increased heart rates and cardiac output remained similar between cancer patients and controls [6]. Reductions in chamber volumes were not accompanied by significant LV mass reductions, which suggests globally decreased loading conditions [6].

**TABLE 2:** Potential culprits of cancer-induced resting sinus tachycardia and underlying pathogenic mechanisms.

Potential factors of cancer-induced resting sinus tachycardia	
Category of insult	Pathogenetic mechanisms
Changes in cardiac output states	Cancer associated anemia Shunting of blood inside tumors Hyperthyroidism Antidiuretic hormone secretion Reduction in LV, RV, and LA volume
Cancer-induced structural heart changes	Lower LV mass Cachexia-associated cardiomyopathy Endothelin-induced changes
Cancer secreted inflammatory cytokines	Systemic inflammatory state
Increased metabolic demands	Hypermetabolic metabolic state Lactic acidosis Anemia Electrolyte abnormalities
Thromboembolic disease	Pulmonary embolism Asymptomatic or silent PE Tumor embolization Pulmonary lymphangitic carcinomatosis
Pain	Stress response Sympathetic responses Psychological stress Anxiety Depression Sleep deprivation Catecholamine release secondary to emotional or physical stress

Abbreviations: LV, left ventricular; RV, right ventricular; LA, left atrial.

Other studies in chemotherapy-naive cancer patients yielded conflicting results regarding LV volume, LV mass, and strain amplitude. These differences can be likely attributed to differences in methodology, as well as different malignancies [7–9]. Interestingly, both heart rate and cardiac output can be reduced with medications such as β-blockers [10]. This makes these agents attractive as potential treatment options for cancer-induced sinus tachycardia. Although they can play a role in reducing the overall cardiac workload, it is unclear whether the above-mentioned changes in cardiac function actually reflect compensatory mechanisms. Hence, whether this increased heart rate is necessary for homeostasis preservation or has a negative effect on the patients' physiology remains unknown.

## Structural Changes

It is well known that cancer can induce structural heart changes. A retrospective study from autopsy reports showed fibrotic changes in the cardiac muscle of cancer patients [11]. On the other hand, it has been shown that the activation of endothelin by breast cancer (and potentially other malignancies) might induce modest cardiac hypertrophy and early left atrial remodeling [12]. These effects were found to be independent of inflammation or fibrosis [12]. Endothelin may also contribute to sinus tachycardia via activation of the sympathetic nervous system [13].

A recent study in 300 patients with advanced, active cancer but without infection or significant cardiovascular disease showed significantly lower LV mass, especially in patients with cachexia [14, 15]. This change in LV mass was independent of previous cardiotoxic anticancer treatment administration, and it was associated with significantly decreased stroke volume, resting tachycardia, and increased 1-year all-cause mortality (43%; 95% CI: 37%–49%) (Table 2). Cardiac muscle wasting was associated with significantly decreased ECOG and Karnofsky performance status, maximum handgrip strength, as well as 6-min walking distance and stair-climbing power [14].

Patients with generalized cachexia were also characterized by significantly decreased blood pressure, which suggests that blood pressure may be useful in the differentiation of cachexia-associated cardiomyopathy from other causes of tachycardia in cancer patients (Figure 1) [14, 16]. Moreover, sinus tachycardia in this case appears to play a compensatory role for the functional decline of cardiac performance. Pre-clinical studies are currently investigating potential therapies for cancer-associated cardiac wasting, with some studies suggesting that bisoprolol, espindolol, or spironolactone may be able to attenuate cardiac wasting [11, 14, 17].

Interestingly, preclinical studies in mice have shown that heart failure, as well as cardiac remodeling without cardiac contractile dysfunction can potentially promote cancer growth and metastasis [18]. Awwad *et al.* used a transgenic mice model to demonstrate the crosstalk between a hypertrophied heart and cancer progression. This communication is believed to be mediated via secreted factors, such as fibronectin, serpin3, or CTgF [18, 19]. These findings suggest that cardiac diseases and cancer may not be completely separate entities, but there might be an underlying connection between these two major causes of morbidity and mortality in humans. Moreover, cancer is notorious for changing the function of normal surrounding or distal cells and tissues, in order to make them work in its favor and promote its progression [20]. The fact that it can alter the structure and function of the entire cardiovascular system to further promote its own growth, only showcases the systemic nature of advanced cancer, and underscores the complexity of its interactions with other body parts.

## Secreted Inflammatory Factors

Cancer induces oncogene-driven cellular processes which result in cellular stress and upregulation of several inflammatory pathways. This is commonly accompanied by increased serum concentration of various inflammatory markers [21]. One can expect that sustained systemic elevation of inflammatory byproducts in the blood may result in a systemic inflammatory-mimicking state, characterized by reduced vascular resistance, tachycardia, increased capillary permeability, and hyper-dynamic contractile state (Table 2) (Figure 1). Cancer-associated cytokines include IL-1, IL-4, IL-6, IL-8, IL-10, IL-17, TNF, interferons, CCL-5, CXCL-1, CXCL-2, VEGF, and FGF, among others [22]. These cytokines not only play a role in cancer progression, but they can also significantly affect cardiovascular function and health [22]. Inflammatory cytokines can directly or indirectly affect cardiac rhythm and promote the development of arrhythmias, and can also affect cardiac remodeling, conferring structural and electrical changes [23, 24]. Inflammatory cytokines can also induce overstimulation of sympathetic signaling, with resulting tachycardia. It has been shown that inflammatory cytokines can interfere with the electrical conduction of the heart, the modulation of membrane ion channels, intracellular calcium ion handling with intracellular overload, and result in gap junction dysfunction, as well as cardiac fibrosis promotion [23, 24].

VEGF has been associated with vasodilation, tachycardia, cardiac output and stroke volume decreases, and both preload and afterload changes [25]. Some cytokines, such as IL-6 have been linked to myocarditis with subsequent myocardial dysfunction [26]. Sinus tachycardia is frequently the first sign of this myocardiopathy. Tachycardia is also a well-known side effect of interferon administration in patients treated for hepatitis C, and constitutes a frequent finding during cytokine-release syndrome [27, 28]. The latter has also been linked with other major cardiac events, such as cardiomyopathy, arrhythmias, and heart failure [28]. These findings underscore the potential of cancer-secreted circulating cytokines to affect cardiac function, and sinus tachycardia is arguably the single most frequent clinical sign associated with these conditions [29].

## Increased Metabolic Demands

Patients with advanced malignancies frequently undergo physiological changes that are characterized by a hypermetabolic state and elevated resting energy expenditure (Table 2). This increased basal metabolic rate is associated with elevated protein catabolism, muscle and fat wasting, weight loss, and hormonal imbalances [30, 31]. Tachycardia is among the most frequent findings in hypermetabolic states, and is considered to be a compensatory mechanism.

The increased burden of rapidly dividing cancer cells requires increased amounts of metabolic fuel to support their growing biomass. Moreover, the end products of their metabolic activities can be toxic compounds [30, 31]. Lactate, the byproduct of anaerobic glycolysis, can result in acidification of the tumor microenvironment, and in advanced cases it may result in lactic acidosis, a life-threatening condition [32, 33].

It has also been suggested that cancer-associated metabolic remodeling can affect the integrity of red blood cells and enhance the ability of the spleen to perform erythrophagocytosis. Together with cancer-associated inflammatory state, it contributes to the anemia that is frequently found in oncological patients, which can also contribute to sinus tachycardia [34]. Moreover, electrolyte abnormalities that can occur due to cancer-associated hypermetabolic state, such as hypomagnesemia, can be proarrhythmogenic and contribute to sinus tachycardia [35, 36].

## Clinical and Subclinical Thromboembolism

Cancer is frequently associated with clinical and subclinical thromboembolic disease [37, 38]. Thromboembolism is an important cause of mortality in patients with malignancies. Cancer by itself activates the coagulation cascade and predisposes to thrombus formation, but frequently other risk factors coexist in these patients, such as prolonged immobilization due to decreased performance status [37, 39]. Tachycardia is one of the most frequent findings in patients with pulmonary embolism [39].

Although venous thromboembolism has been extensively studied, arterial thrombosis is also more frequent in cancer patients compared to controls [40]. Other thrombotic complications of cancer also exist, such as chronic disseminated intravascular coagulation and thrombotic microangiopathy [38]. Factors that may further increase the risk of thrombotic events include hospitalization, chemotherapy, antiangiogenic factors, and the placement of central venous catheters for drug delivery [38].

Although pulmonary embolism is a dramatic complication of venous thromboembolism, studies have shown that there are also cases of asymptomatic or silent pulmonary embolism [39] (Table 2). Older series have shown that the incidence of silent pulmonary embolism can reach up to 70% in high-risk patients [41]. Hence, one can hypothesize that several cases of unexplained sinus tachycardia in patients with advanced malignancies can be attributed to undiagnosed thromboembolism of small peripheral branches of pulmonary arteries. Moreover, undiagnosed embolic events in other parts of the body can easily go unrecognized in patients with advanced cancer, as symptoms of pain can be attributed to the cancer itself and typical biomarkers, such as d-dimers are less diagnostic and specific in cancer patients. These events can be the cause of a systemic inflammatory reaction, which can manifest with various symptoms, including sinus tachycardia.

In addition, cancer patients may experience tumor embolization, which occurs when tumor emboli translocate and occlude small vessels, with resulting ischemic events. Pulmonary lymphangitic carcinomatosis is also a rare condition which can lead to pulmonary hypertension and right heart failure in small subsets of patients with disseminated malignancies [42].

## Pain

Cancer-associated pain is one of the most devastating symptoms cancer patients experience. The severity of pain frequently requires the use of opioids for management [43].

Although the exact degree of association is still debated, severe pain is classically considered to elicit a stress response characterized by sinus tachycardia, as well as elevated blood pressure [44] (Table 2). Studies in preclinical models revealed that painful stimuli may trigger sympathetic responses, which may affect heart rate [45]. Moreover, cancer patients may be in constant psychological stress, which might be related to copying with the cancer diagnosis itself, or with cancer treatment. Cancer patients frequently are experiencing anxiety, depression, while they frequently suffer from sleep deprivation. Emotional or physical stress can induce catecholamine release, which can promote sinus tachycardia, increased blood pressure, and stress hormone release [46].

## Cancer Therapeutics

Several cancer treatments have been associated with cardiac adverse effects that can clinically manifest as resting sinus tachycardia. Anthracyclines, including doxorubicin and idarubicin, frequently result in dose-dependent decreases in left ventricular function. Although many times the effects are subclinical, in severe cases they may result in congestive heart failure [47]. Taxanes, such as paclitaxel, have also been associated with ejection fraction reductions, especially in patients with high risk features, including elderly, hypertensive or diabetic patients, or patients that have previously received radiotherapy to the chest wall [48]. Fluoropyrimidines are among the most cardiotoxic chemotherapeutic agents and several pathophysiological mechanisms have been proposed, such as endothelial damage, toxic metabolites, oxidative stress, Krebs cycle disturbances, or coronary vasospasm. These result in a wide range of cardiotoxic effects, many of which can manifest as sinus tachycardia [49]. Cardiotoxicity, including severe myocarditis, is a well known side effect of cyclophosphamide as well [50]. Platinum-based agents also carry cardiotoxic potential due to direct toxicity to cardiomyocytes, or formation of reactive oxygen species with subsequent inflammation and thrombus formation [51].

Moreover, newer anticancer agents have been linked to adverse cardiac effects, which can manifest as resting sinus tachycardia. Human epidermal growth factor receptor 2 (HER2) inhibitors carry cardiotoxic potential in a subset of patients. For example, trastuzumab toxicity can manifest as left ventricular dysfunction with a decline in ejection fraction to 10%–15% in about 9% and ≥16% in about 2% of patients in 1 year. Although most cases of cardiotoxicity are asymptomatic and reversible with treatment discontinuation, a small percentage can be symptomatic or even persistent after treatment discontinuation [52, 53]. Immune checkpoint inhibitors have also been rarely associated with immune-mediated myocarditis or pericarditis. They have also been associated with various arrhythmias, including sinus tachycardia, which are believed to be mediated by local inflammation to the conduction system. Although arrhythmic events are considered rare, they can occasionally be life-threatening [54, 55].

## Recommendations/Future Perspectives

Resting sinus tachycardia is frequently associated with cancer, highlighting the systemic nature of the disease. Although it can be a sign of an underlying condition that needs immediate attention, such as pulmonary embolism, bleeding or sepsis, it can also be a primary sign of advanced cancer and no further testing is needed, depending on the patient’s goals of care. There is currently no specific test to reliably diagnose cancer-associated resting tachycardia, thus it should be a diagnosis of exclusion. The lack of extensive dedicated literature on the condition often makes it an “uncomfortable” diagnosis, especially for patients without advanced stage cancer. Although not immediately life-threatening it has been associated with worse outcomes. However, it is unknown if this is due to the fact that sinus tachycardia is a hallmark of systemic response that signals a more aggressive cancer, or if the tachycardia has a negative impact on survival *per se*. As a result, many clinicians attempt to lower the resting heart rate, e.g., with low dose beta-blockers, especially for severe resting sinus tachycardia (>120 beats per minute). However, the pathogenetic mechanisms might not be universal and each case may require a different approach.

Significant resting sinus tachycardia can be either a compensatory mechanism or an adverse effect of cancer. Although treating the latter case may be beneficial, treating the former case can theoretically be detrimental. Although there is no gold standard to differentiate these two cases, some signs can strongly suggest one or the other etiology. Examples include a lower or higher blood pressure than baseline, structural and functional findings in echocardiography and other cardiac imaging modalities, pH and lactate levels in the blood, a very high level of d-dimers, the presence of significant pain, generalized wasting, etc. The development of a scoring system to estimate the likelihood that sinus tachycardia is compensatory carries the potential to be useful to guide therapeutic decisions in the future. Further research is needed to identify the optimal management for cancer patients that may improve survival and quality of life, in addition to their direct cancer therapies.

## Conclusion

Cancer-associated resting sinus tachycardia is an understudied condition with uncertain clinical significance. Here, we attempted to bridge the gap in the literature and provide the foundation for further research to improve our understanding of the pathophysiology, as well as to improve diagnostic and therapeutic management of cancer-associated resting sinus tachycardia.
